# Treatment of posttraumatic patella osteomyelitis with MRSA infection and knee stiffness: a case report

**DOI:** 10.1186/s12893-020-00996-1

**Published:** 2020-12-07

**Authors:** Haiyong Ren, Kai Huang, Peijian Tong, Yansheng Zhu

**Affiliations:** 1 Department of Orthopedics, Tongde Hospital of Zhejiang Province, 234 Gucui Road, Hangzhou, 310012 Zhejiang China; 2 Department of Orthopaedic Surgery, The First Affiliated Hospital of Zhejiang Chinese Medical University, Hangzhou, 310006 Zhejiang China

**Keywords:** Patella, Posttraumatic osteomyelitis, MRSA, Knee stiffness, Ilizarov frame, Gastrocnemius muscle flap

## Abstract

**Background:**

Posttraumatic patella osteomyelitis is rare, and the treatment of osteomyelitis remains to be challenging. Control of the infection commonly costs a long time, and it is easily to cause knee stiffness. In addition, there is no unified protocol for the treatment of knee stiffness.

**Case presentation:**

We reported a case of posttraumatic patella osteomyelitis that successive infected with methicillin-resistant staphylococcus aureus (MRSA) after multiple surgeries. We successfully treated osteomyelitis by one-staged surgery, but the patient present knee stiffness after treatment. Thus Ilizarov external fixation system was further used to gradually adjust the mobility by exerting mechanical stress to the joint. After adjusting the frame under a scheduled plan, the patient successfully restored satisfactory knee function.

**Conclusions:**

Adequate debridement is the key to control infections of posttraumatic osteomyelitis. Control the infection of posttraumatic patella osteomyelitis by one-staged surgery is achievable and could shorten the knee immobilization period. When knee stiffness occurs, scheduled range of motion (ROM) adjustment using Ilizarov frame with hinges might be a safe and useful method to restore function.

## Background

Osteomyelitis remains to be a challenging disease to manage [1, 2]. Post-traumatic osteomyelitis is mostly resulted from open fractures or improper management during surgical treatment, while posttraumatic osteomyelitis of the patella is relatively rare [3]. Treatment of posttraumatic patella osteomyelitis may be complicated by conditions such as tissue defects, fracture nonunion, ruptured patellar ligament or quadriceps tendon, or deep knee joint infection [4, 5]. The treatment commonly costs a long time; it is generally hard to achieve early rehabilitation and is easily to cause knee stiffness. Here we report one case of posttraumatic patella osteomyelitis with MRSA infection. The patient also suffered knee stiffness after long time immobilization. We controlled the infection by one-staged surgery, and Ilizarov external fixation was used to successfully restore the knee function.

## Case presentation

A previously healthy 31 years old man was transferred to our hospital with history of closed fracture of the right patellar after fall injury 13 months ago. He was treated surgically with open reduction tension band wiring at a local hospital. The patient developed wound infection after surgery. The wound was debrided 3 times in the following half a year and he also received long term antibiotic treatment, but no improvement was achieved.

**Fig. 1:**
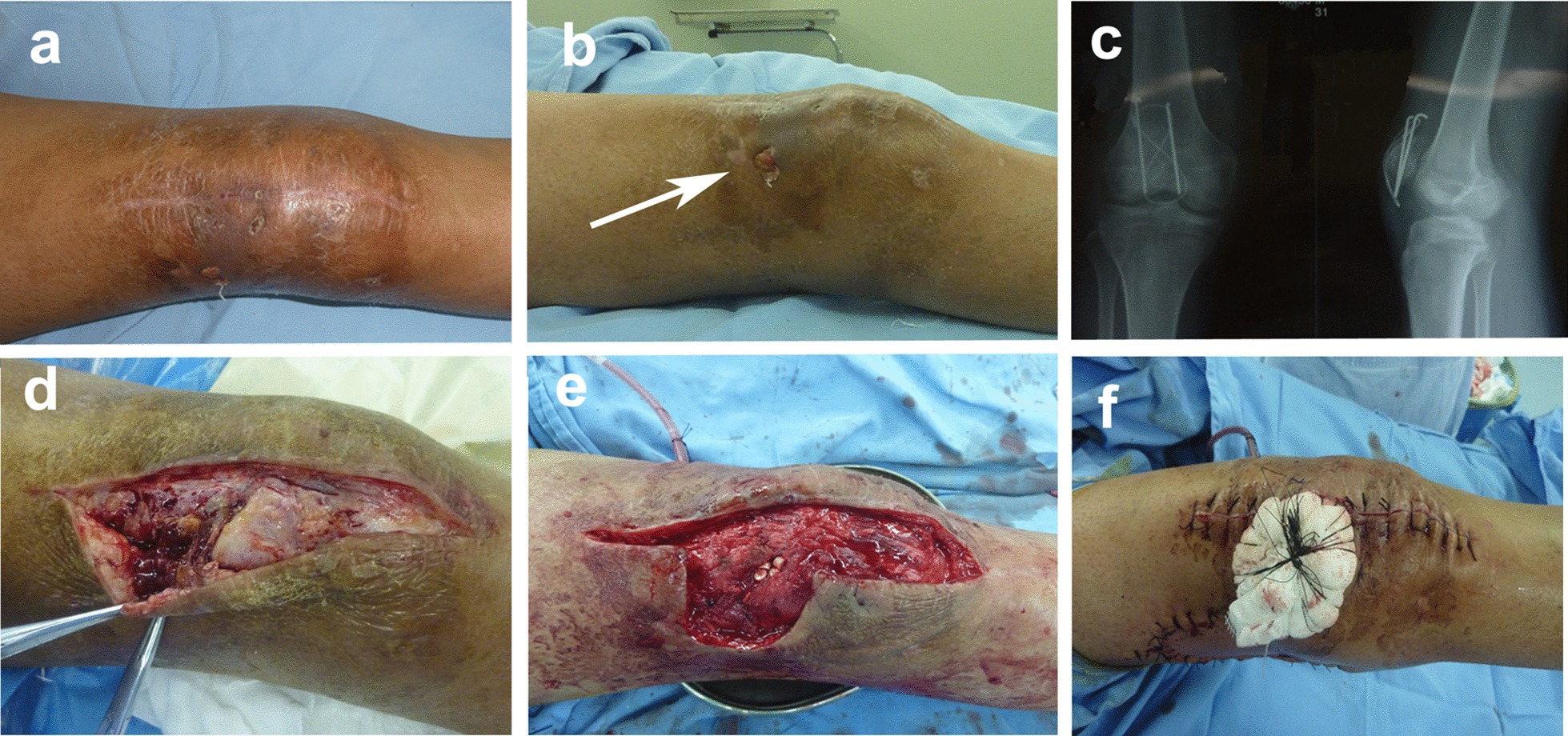
**a**, **b** Appearance of the right knee at the time of admission, and the arrow indicates infected sinus; **c** frontal and lateral radiographs of right patella at the time of admission; **d** infected bone and soft tissues were observed during operation; **e** intraoperative appearance after thorough removal of infected bone and soft tissues, and cavities were implanted with RBK beeds; **f** wound repaired by lateral gastrocnemius flap and skin graft

On admission, the physical examination revealed normal body temperature. Mild swelling and normal skin surface temperature of the right knee was observed. A 10 × 5 mm sinus tract was located close to patella, and the range of motion (ROM) of the knee was 0°–40° (Fig. 1a, b). Bacterial culture of the exudations at the sinus was MRSA. Laboratory results showed white blood cell count (WBC) was 7.3 × 10^9^/L, C-reactive protein was 10.8 mg/L, and erythrocyte sedimentation rate was 56 mm/h. The radiograph showed the wires had loosened (Fig. 1c). Synovial fluid of the knee was obtained by aseptic puncture. The fluid culture was negative and fluid analysis showed the WBC was less than 1000/mL, which suggested no joint infection [6].

Surgical debridement was performed 4 days after admission to our hospital. Necrotic and inflammatory tissues were found in the deep end of sinus tract and were very close to the wires (Fig. 1d). The abnormal tissues were completely excised. The wires were removed, and the tracts of the wires were thoroughly debrided with a curette. After washing the wound with a large amount of saline, the debrided cavity and pin tracts were filled with vancomycin-loaded synthetic calcium sulfate beads (Osteoset Resorbable Kit, RBK, Wright Medical, Arlington) (Fig. 1e). The lateral gastrocnemius myocutaneous flap was dissected and transferred to fully cover the patella. The knee was kept in extension position and a drain tube was placed under the muscle flap. Split-thickness skin grafts were grafted on the muscular flap and then compressed with a gauze pad (Fig. 1f).

**Fig. 2:**
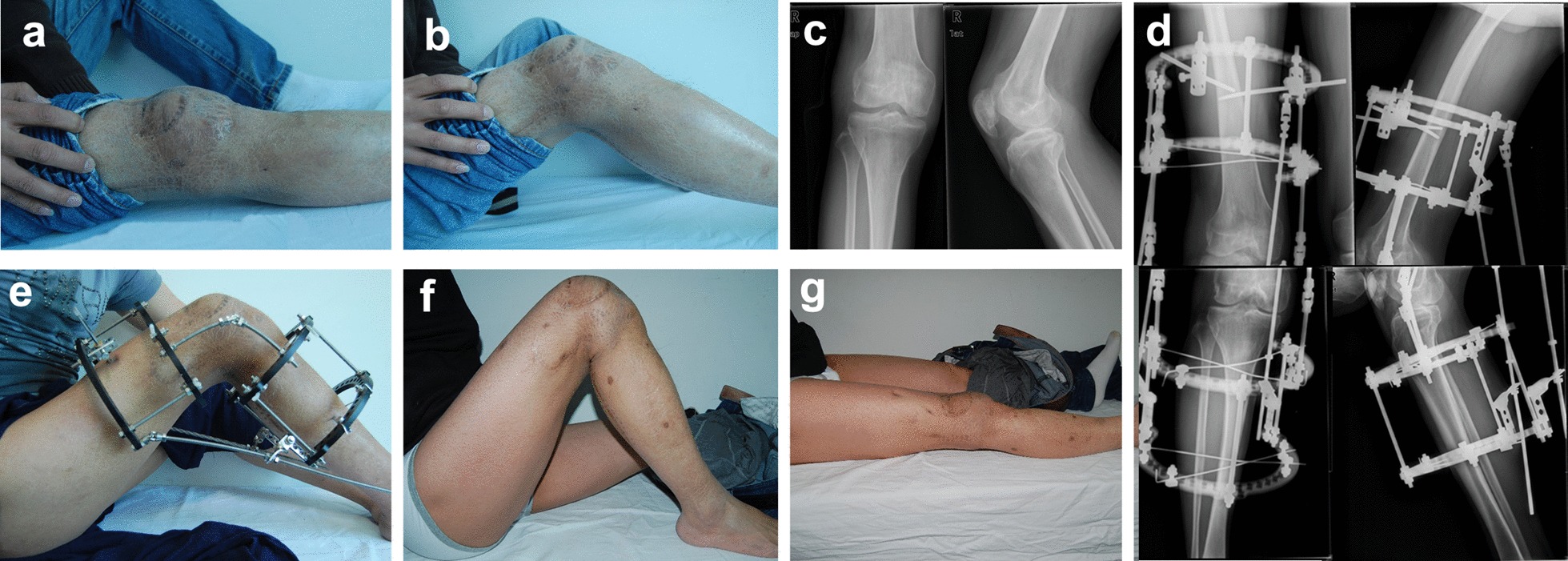
**a**, **b** Right knee ROM was 0°–40° at 1 year after first surgery; **c** no bone destruction observed in radiograph at follow-up; **d** frontal and lateral radiographs of right knee after fixation with a Ilizarov frame; **e** Ilizarov ring external fixation was implanted into the right tibia and femur, with the external fixation connected by hinges; **f**, **g** follow-up at 7 weeks after external fixation removal, right knee maintained ROM of 0°–130°

After initial surgery in our hospital, the knee was immobilized with a straight brace for 2 weeks. The patient received intravenous linezolid treatment. One week after initial surgery, the gauze pad was removed and the skin grafts showed well-healed, meanwhile the drain tube was removed. Laboratory inflammation markers were normal at 3 weeks after surgery and the patient was discharged. The patient continued oral linezolid therapy for 4 weeks after discharge. Active and passive motion exercise of the right knee was also instructed. No sign of infection was observed over the following 1 year (Fig. 2a, b). Radiograph at follow-up showed no bone destruction of patella (Fig. 2c). However, ROM of the right knee was limited to 0°–40°.

The patient claimed difficulty with climbing stairs and squatting due to right knee stiffness. No significant improvement was achieved after therapy for about 1 year with guidance from a rehabilitation physician. The patient therefore appealed for further treatment and rehospitalized 1 year after the last discharge from our hospital. Ilizarov external fixation was chosen to manage the knee stiffness. An Ilizarov external ring fixator consisting of four rings and several K-wires was placed on the distal femur and proximal tibia. Hinges were placed between the external fixators. The most proximal and distal rings were connected with two long threaded rods with nuts. The angles of the hinges were adjusted by rotating the nuts on the threaded rods (Fig. 2d, e). A scheduled plan was made to adjust the angle of the knee. We flexed the right knee to the fullest and further turned the nuts 5 mm to the proximal end on the first day morning (8 am) to exert persistent bending force on the knee. The locations of the nuts on the threaded rods were marked. The knee was returned to the strait position before sleep (9 pm). The next day morning, the knee was flexed and the nuts were turned to 2.5 mm more proximal than the previous marked location. After adjusting the knee motility for two months, ROM of the right knee reached 0°–120°. Then the fixators were removed. After removal of the external fixation, the patient performed further active and passive knee motion exercises. The last follow-up visit was 2 months after removal of the fixators. Laboratory inflammation markers were normal. The knee presented normal appearance and ROM of the joint was 0°–130°. The patient claimed no pain and no obvious obstacles when climbing stairs or squatting (Fig. 2f, g).

## Discussion

Posttraumatic patella osteomyelitis is rare. At present surgical treatment remains the most important therapy of posttraumatic osteomyelitis [7]. Surgical treatment requires thorough debridement of infected or necrotic tissues and may easily cause soft tissue or bone defects. Inexperienced surgeons might be perplexed about repairing large tissue defects and would be more likely to debride incompletely. Residual pathological tissues would cause persistence of infection or further result in knee joint infections. That might also be the reason that our case failed to control the infection after multiple surgeries at the local hospital. In our case, necrotic and inflammatory tissues were found in the deep end of sinus tract, and the abnormal tissues were close to the wires. It is well-know that bacterial adhesion and biofilm formation on implant surfaces could shelter the bacteria and encourage persistence of infection [8]. So we removed the wires and performed extensive debridement. In addition, repeated surgery and prolonged immobilization of the knee would worsen knee stiffness. As the patella locates superficially, the bone would easily be complicated with lack of tissue coverage after trauma, surgery or infection. When sufficient debridement is achieved, the wound is better to be repaired at the same operation stage. Plastic techniques or microvascular skills could play important roles in repairing tissue defects. Flaps could repair the tissue defect and provide sufficient local blood supply, which further promote bone healing and control of infection [9]. In complex conditions involving the quadriceps or patellar tendon defects, the gastrocnemius myotendinous flap can be used to reconstruct the tendon and repair the tissue defect simultaneously [10]. Additionally, antibiotic-loaded carriers could sustained releasing anti-infective agents directly at the local site of infection and facilitate infection control [11, 12]. We controlled the infection and repaired the wound through single-staged surgery, multiple surgeries were avoided and the duration of knee immobilization was largely shortened.

Postoperative stiffness is a debilitating complication [13, 14]. As to our case, anterior adhesions or retractions (joint capsule, quadriceps bursa, quadriceps) were considered the primarily reasons leading to knee stiffness, while the impaired articular surface of patella might be partly responsible for the dysfunction. Various therapeutic methods could be chosen to manage knee stiffness, including manipulation under anesthesia, arthroscopic arthrolysis, open quadriceps release and so on [7, 14]. However, there is no widely accepted protocol for its treatment. Because of the previous posttraumatic patella osteomyelitis, arthroscopic or open surgeries were not preferred for fear of recurrence of infection. Meanwhile, the lateral gastrocnemius myocutaneous flap had been transferred to the anterior knee, the altered structure of the knee would increase the operating difficulty and postoperative complication risk. The Ilizarov method remains to be one of the important contributions to the field of orthopaedics [15]. Today it is widely used for reconstruction of nonunions, management of bone defects, deformity correction and limb lengthening. By lengthening over an external fixator, distraction osteogenesis was achieved along with a lengthening of tendons, muscles, nerves and vessels at the same time [16]. Ilizarov external fixation system is also an effective way to improve joint mobility. Previously, some researchers had successfully treated cases of flexion contraction of elbow, knee, or ankle joint by using the Ilizarov external fixator [17–20]. The external fixators exert persistent mechanical stress to the bone while lengthening and reshaping the tissues according to the law of tension-stress [21]. Meanwhile it could avoid invasive damage to the surrounding blood vessels and nerves. Adjusting joint mobility by the Ilizarov frame could avoid invasive procedures and reduce related risks. But the effect of Ilizarov frame on joint stiffness or flexion obstacles still lacks of sufficient clinical evidence. Our case indicated that Ilizarov external fixation system might also be safe and useful for management of joint flexion limitation. Invasive procedures can be avoided, and by adjusting the frame with a scheduled plan, persistent bending forces were exerted on the knee to gradually remodel the tissue structures. The regime of repeated flexion and extension of the joint every day could better promote joint function restoration. During the treatment, the lower limbs could still bear weight due to the stability of the external fixators. In our case, no “rebound phenomenon” of the joint was observed at the follow-up [18, 22].

## Conclusions

We believe that adequate debridement is the key to control infections of osteomyelitis. Possessed with plastic techniques or microvascular skills could help surgeons dispel the hesitations to perform sufficient debridement. Meanwhile, use of flaps and implantation of antibiotic-releasing carriers, as well as choosing suitable antibiotics could all play important roles in osteomyelitis treatment. Adequate debridement and repairmen of the wound by one-staged surgery could shorten the knee immobilization time. When knee stiffness occurs, scheduled ROM adjustment using Ilizarov frame with hinges could gradually remodel the tissue structures without invasive procedures, which might be a safe and useful method to manage joint stiffness.

## Data Availability

All data generated or analysed during this study are included in this published article.

## Abbreviations

MRSA: Methicillin-resistant staphylococcus aureus
ROM: Range of motion
WBC: White blood cell count
